# Validation of GeneFinder COVID-19 Ag Plus Rapid Test and Its Potential Utility to Slowing Infection Waves: A Single-Center Laboratory Evaluation Study

**DOI:** 10.3390/diagnostics12051126

**Published:** 2022-05-01

**Authors:** Cinzia Peronace, Rossana Tallerico, Manuela Colosimo, Vanessa Sacco, Roberta Talarico, Marco De Fazio, Federica Pasceri, Ilenia Talotta, Giuseppina Panduri, Jung-Hee Kim, Erika Cione, Pasquale Minchella

**Affiliations:** 1 Microbiology and Virology Unit, Pugliese-Ciaccio Hospital, 88100 Catanzaro, Italy; rossana.tallerico@gmail.com (R.T.); manuelacolosimo@hotmail.it (M.C.); vanessasacco22@gmail.com (V.S.); robytalarico@yahoo.it (R.T.); marco_2592@yahoo.it (M.D.F.); federicapasceri@gmail.com (F.P.); ilenia.talotta@gmail.com (I.T.); pandurigiuseppina@gmail.com (G.P.); pminchella@aocz.it (P.M.); 2 Department of Neurosurgery, Seoul Medical Center, Seoul 02053, Korea; nostoi72@naver.com; 3 Department of Pharmacy, Health, and Nutritional Sciences, University of Calabria, 87036 Rende (CS), Italy

**Keywords:** COVID-19, early diagnosis, rapid antigen detection test, variants of concern

## Abstract

Diagnostic laboratory tools are essential to keep everyone safe and track newly emerging variants; on the other hand, “filter” screening tests recognizing positivity are valuable tools to avoid hectic laboratory work that, besides COVID-19, are also part of the routine. Therefore, complementary assays, such as rapid antigen tests (RATs), are essential in controlling and monitoring virus spread within the community, especially in the asymptomatic population. A subset of nasopharyngeal swab specimens resulted in SARS-CoV-2 positive and investigated for genomic characterization were used for RAT validation. RATs were performed immediately after sampling, following the manufacturer’s instructions (reading at 15 min). RT-PCRs were carried out within 24 h of specimens’ collection. Out of 603 patients, 145 (24.05%) tested positive by RT-PCR and RAT and 451 (74.79%) tested negative by both methods; discordant results (RT-PCR+/RAT− or RT-PCR−/RAT+) were obtained in 7 patients (1.16%). RATs’ overall specificity and sensitivity were 96.03% (95%CI: 91.55–98.53%) and 99.78% (95%CI: 98.77–99.99%), respectively, taking RT-PCR as the reference. Overall, RAT negative predictive value was 98.69% (95%CI 97.17–99.40%). The GeneFinder COVID-19 Ag Plus Rapid Test performed well as a screening test for early diagnosis of COVID-19, especially in asymptomatic subjects. The data suggested that patients with RT-PCR-proven COVID-19 testing negative by RAT are unlikely to be infectious. GeneFinder COVID-19 Ag Plus Rapid Test also works on variants of concern (VOC) delta and omicron BA.1 and BA.2.

## 1. Introduction

Since the epidemic’s beginning on 6 March 2020, the integrated surveillance system counted 12,737,375 cases, of which 152,260 deaths [1] have been diagnosed from coronavirus disease in 2019 (COVID-19). With numbers so high that are not easy to control and to counteract virus spread, to date, Real-Time RT-PCR (RT-PCR) is the standard gold method to diagnose SARS-CoV-2 acute infection [2]. Diagnostic laboratory tools are essential to keep everyone safe and track newly emerging variants. However, complementary assays, such as rapid antigen tests (RATs), have been developed by several biotech companies to control and monitor virus spread within the community [1]. Unfortunately, in the past 30 days, the Calabria region has not been able to timely report all cases diagnosed. Therefore, we must consider that the presented data are affected by some degree of under-notification [2,3]. The missing data on the infection waves in our region do not permit infection control to know if infection is higher or lower or in balance. Therefore, there is an urgent need to have a reliable and rapid screening tool. This study is the first to evaluate GeneFinder COVID-19 Ag Plus Rapid Test (OSANG Healthcare Co., Ltd. Anyang, Gyeonggi, Korea). A subset of nasopharyngeal (NPH) swab specimens that resulted in SARS-CoV-2 positive and investigated for genomic characterization were used for GeneFinder COVID-19 Ag Plus Rapid Test validation. The performance of this lateral flow immunoassay was compared with the SARS-CoV-2 RT-PCR for viral gene detection assay, Allplex™ 2019-nCoV Assay (Seegene^®^, Seoul, Korea). Furthermore, we divided specimens into three groups: symptomatic hospitalized (SH) and non-hospitalized patients (NH) at the onset of symptoms, with a subgroup of asymptomatic non-hospitalized (ANH) from the latter group.

## 2. Materials and Methods

### 2.1. Patients and SARS-CoV-2 Testing

The samples were collected in October 2021 from patients admitted to the Emergency Unit of Pugliese-Ciaccio Hospital of Catanzaro with respiratory problems and symptoms indicative of COVID-19 with or without pneumonia such as fever, loss of taste or smell, or shortness of breath or cough. Samples were also collected from subjects who required molecular testing and others who were asymptomatic but had a contact with a confirmed case of COVID-19 during the past ten days. RATs were performed immediately after sampling. Reverse Transcription-Polymerase Chain Reaction (RT-PCR) was carried out within 24 h of specimen collection in real-time mode. With this approach in the study, we enrolled 603 patients with clinical suspicion of COVID-19 in symptomatic hospitalized (SH) individuals who had respiratory failure (*n* = 32) and non-hospitalized subjects (NH), referring to a drive-in testing facility (*n* = 119) of which there were 14 asymptomatic non-hospitalized (ANH) ones. Symptomatic hospitalized patients (SH) and asymptomatic non-hospitalized (ANH) underwent NPH swabs collected in a Universal Transport Medium (UTM™) Copan tube and were tested for this purpose. Molecular diagnosis of SARS-CoV-2 infection was performed with Automated Liquid Handling Workstations NIMBUS for nucleic acid extraction and the Bio-Rad CFX96™ Dx Real-Time PCR System (Hercules, CA, United States) using the Allplex™ 2019-nCoV Assay (Seegene^®^, Seoul, Korea) kit. A cycle threshold value (Ct value) < 40 for all four target genes detected (E, RdRp/S and N genes) was defined as a positive result.

### 2.2. GeneFinder COVID-19 Ag Plus Rapid Test Assay

The GeneFinder COVID-19 Ag Plus Rapid Test, based on an immunochromatographic assay was carried out according to the manufacturer’s instructions. Briefly, 100 µL of UTM™ was dropped in the GeneFinder COVID-19 Ag Plus Rapid Test card and then it was read at 15 min. The nucleocapsid proteins from SARS-CoV-2 are recognized by capturing antigen-conjugate gold particle complexes. They migrate across a reaction area coated by antibodies to nucleocapsid proteins. Positive results display two colors related to control (C) and test (T) lines, while only one line in the C area is present for the negative one. UMT™ blanks or negative UTM™ pools did not give false-positive results (data not shown). A Cut-off-Index (COI) is calculated using pixel number as the ratio between test and control bands. To evaluate the cross-reactivity of the RAT test, positive NPH swabs for non-SARS-CoV-2 respiratory virus isolates were also assessed by molecular assay. The other viruses inducing respiratory disease were Adenovirus [4], Coronavirus (229E, HKU1, NL63, OC43) [5], Human Metapneumovirus [6], Human Rhinovirus/Enterovirus [7], Influenza A, Influenza A H1-2009, Influenza A H3 [8], Influenza B, Parainfluenza types 1,2,3,4 [9], Respiratory Syncytial Virus A, B, *Chlamydia pneumoniae* and *Mycoplasma pneumoniae* [10].

### 2.3. Sequencing SARS-CoV-2

Automatic KingFisher Duo Prime by Thermo Fisher (Waltham, MA, USA) was used to extract SARS-CoV-2 nucleic acid by briefly extracting a 200 μL aliquot of specimen in UTM™ using the MagMAX™ Viral/Pathogen Nucleic Acid isolation kit on the KingFisher Flex Purification system (Waltham, MA, USA). Moreover, a subset of SARS-CoV-2 positive swabs investigated for genomic characterization via next-generation sequencing (NGS) was also tested by GeneFinder COVID-19 Ag Plus Rapid Test. The NGS approach by MiSeq System (Illumina, San Diego, CA, USA) provided 2 × 250 bp read length data. The SOPHIA DDM Platform analyzed FASTQ reads. Clade analysis was obtained by ICOGEN Platform. Then, lineage information was described using the Pangolin nomenclatures as previously described [11,12].

### 2.4. Ethics

The study has been approved by the Ethical Committee Central Area Zone Calabria (reference number #352, 21 October 2021). The performance of examinations complied with the specifications of the Declaration of Helsinki.

### 2.5. Statistical Analyses

Agreement between RATs and RT-qPCR results were calculated using Cohen’s κ coefficient. A receiver operating characteristic curve (ROC) was generated to provide another assessment for the diagnostic power of the RATs. These analyses were done using GraphPad Prism version 8.0.0 for Windows, GraphPad Software (San Diego, CA, USA, at www.graphpad.com).

## 3. Results

During the study period, 603 samples were tested for COVID-19 with rapid antigen tests and Real-Time RT-PCR assays. Of these, 24.05% (*n* = 145) were resulted positive for SARS-CoV-2 NAAT and RAT, while 74.79% (*n* = 451) were resulted negative by both methods; discordant results were obtained in 1.16% (*n* = 7), of which six false-negative results were obtained from samples with Real-Time RT-PCR cycle threshold Ct >28; only in one case, a cross-reactivity was recorded for the most common respiratory viruses, the Respiratory Syncytial Virus (RSV) (Table 1).

Comparing GeneFinder COVID-19 Ag Plus Rapid Test to RNA detection by Real-Time RT-PCR assay, the sensitivity, specificity and overall percent agreement of rapid SARS-CoV-2 antigen tests were 96.03% (145/151; CI: 91.55–98.53%), 99.78% (451/452; CI: 98.77–99.99%) and 98.84% (596/603; CI: 97.62–99.53%), respectively. Predict positive value (PPV) and predict negative value (NPV) were found at 99.32% (95% CI: 95.43–99.90%) and 98.69% (95% CI: 97.17–99.40%). Cohen’s Kappa was 96.87 (SE:0.01, 95% CI: 0.9457–0.9918), as reported in Table 2.

The NPHs were subjected to 40 cycles of RT-PCR amplification. RT-PCR Ct < 25 best discriminated between RT-PCR+/RAT+ and RT-PCR+/RAT– specimens amongst groups SH, NH and ANH, respectively, with a sensitivity and specificity of 100% (Table 3).

**Figure 1:**
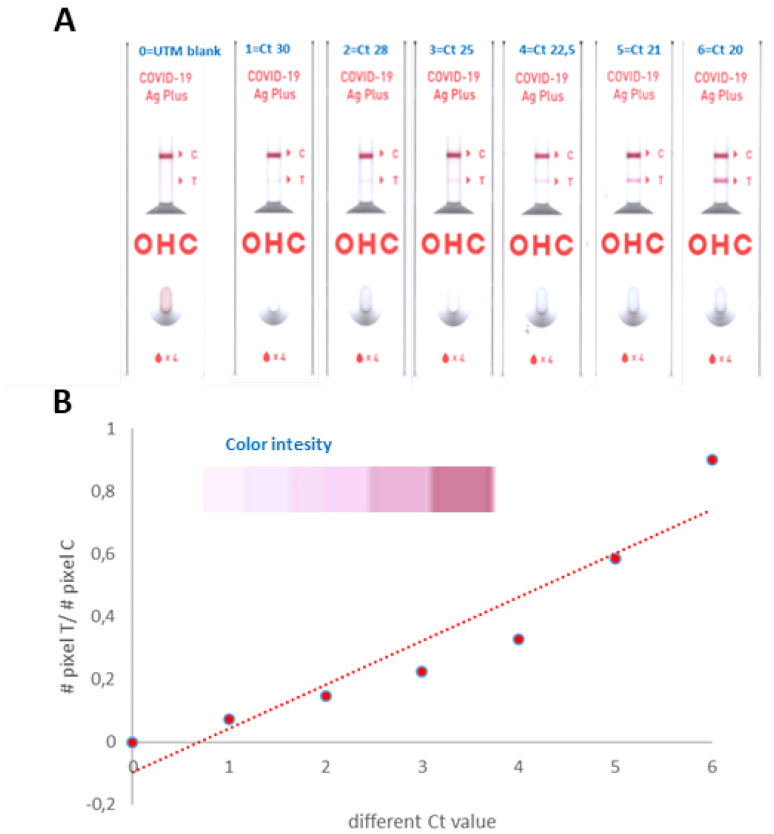
(**A**) RAT test with different Ct values and (**B**) COI index.

**Figure 2:**
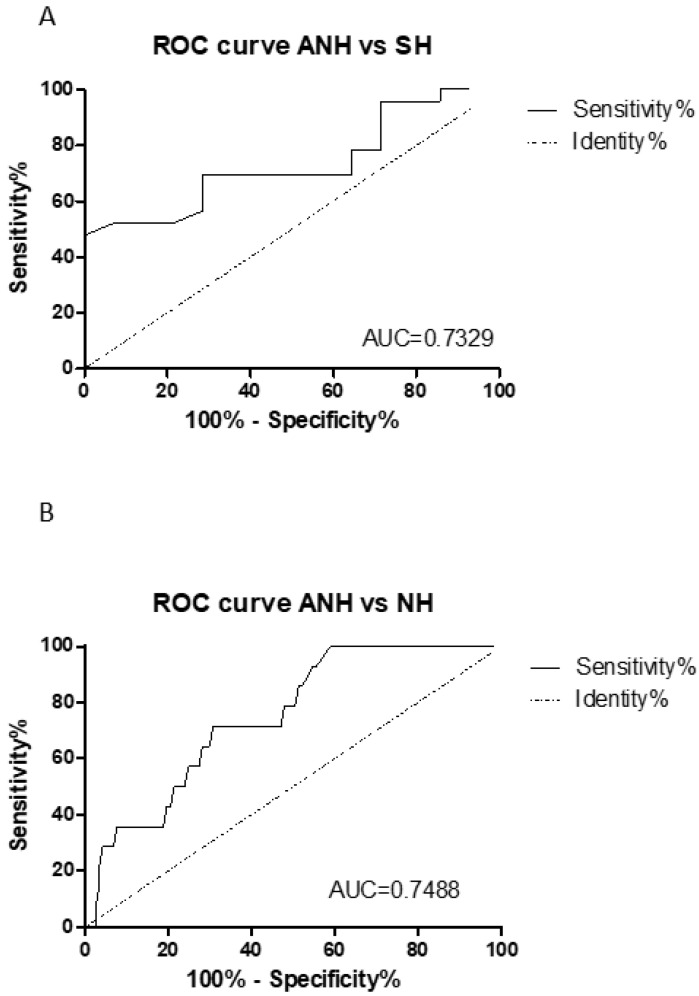
ROC curve analysis (**A**) ANH and SH; (**B**) ANH and NH.

Cut-off indices (COI) are calculated as the ratio between pixel test and pixel control bands using Photoshop to read the average of the pixel area on the lateral immunochromatography test and can be considered a measure of signal strength. The limit of detection (LOD) was assessed with different NPH swabs with Ct value between 20 to 36 for N gene (Figure 1A). For Ct equal to 36, the RATS resulted in false-negative (data not shown). UTM buffer (blank) does not interfere with the antibody/antigen complex formation. To link band intensity from RATs to Ct value, we also noticed a different color intensity (Figure 1B). COI was used for receiver operating characteristic (ROC) curve analysis to evaluate the predictive power of RAT+ deriving from Ct value. This results in an AUC of 0.7329 for ANH and SH specimens and an AUC of 0.7488 for ANH and NH (Figure 2A,B, respectively).

Discordant results (RT-PCR+/RAT− or RT-PCR−/RAT+) were obtained in 7 samples (1.16%), of which 6 false-negative results were obtained from samples with a high Real-Time RT-PCR cycle threshold Ct >28. Only in one case was a cross-reactivity result for one of the most common respiratory viruses (Respiratory Syncytial Virus A/B) found.

Moreover, we are also reporting the capability of GeneFinder COVID-19 Ag Plus Rapid Test to recognize variants of concern. The RAT gave positivity for 9 Delta clades 21K (*n* = 3), 21J (*n* = 3), 21A (*n* = 1) and 21I (*n* = 2), 3 Omicron BA.1 clades 21K and 2 Omicron BA.2 clades and 21L (Table 4). We considered only mutations in N gene protein. In particular, we found the following mutations linked to specific lineages, to Delta VOC: (i) D63G; G215C; D377Y for B.1.617.2; (ii) D63G; R203M; G215C; D377Y for AY.4; (iii) A35; D63G; R203M; G215C; D377Y for AY.39; (iv) A35; D63G; R203M; G215C; D377Y for AY.42; (v) A35; D63G; L139F; R203M; G215C; D377Y for AY.122; (vi) D63G; R203M; G215C; D377Y for AY.46.6; (vii) D63G; R203M; S327; D377Y; D399Y for AY.53; (viii) D63G; T135I; R203M; D377Y for AY.61; (ix) D63G; R203M; D377Y for AY.9.2 and Omicron VOC: (i) P13L; GERS30G_del; R203K; G204R for B.1.1.529; (ii) P13L; GERS30G_del; R203K; G204R for BA.1; (iii) P13L; GERS30G_del; R203K; G204R for BA.1.1; (iv) P13L; GERS30G_del; R203K; G204R; S413R for BA.2; (v) P13L; GERS30G_del; R203K; G204R; S413R for BA.2.

## 4. Discussion

Real-Time RT-PCR assays for SARS-CoV-2 RNA detection in clinical specimens are widely used in COVID-19 diagnostic laboratories as the standard gold method. These require dedicated instruments and operator expertise to conduct RT-PCR assays. It was also reported that, in effect, the screening depends largely on frequency of testing and speed of reporting and is only marginally improved by high test sensitivity even in the acute infection [13,14,15]. Therefore, rapid and accurate tests for SARS-CoV-2 screening are essential for surveillance of virus spread. In this scenario, lateral flow immunoassays using monoclonal anti-SARS-CoV-2 antibodies, which target SARS-CoV-2 antigens, mostly nucleocapsid protein, can be complimentary screening tests. This is ideal if their accuracy is comparable to the molecular results obtained by RT-PCR. An important aspect in the pandemic, even if we are entering an endemic condition, is the auto-surveillance. The rapid and continuous detection efforts aimed at early recognition, isolation and treatment of infected people are still crucial for slowing down infection waves [9]. Based on the real-world data described here, the commercially available rapid SARS-CoV-2 antigen detection kit (GeneFinder COVID-19 Ag Plus Rapid Test) was compared with the RT-PCR Assay (Allplex™ 2019-nCoV Assay) for detection of SARS-CoV-2 infection. The sensitivity and specificity of several commercial rapid antigen tests are reported in the literature [3,4,5,6,7,8]. Compared to RT-PCR, the GeneFinder COVID-19 Ag Plus Rapid Test showed an excellent specificity as evidenced by ROC curves and an excellent sensitivity, respectively (98.84% and 99.78%); the latter improved when the testing was done less than five days from the onset of symptoms (100%). By our choice, we included a significant fraction of specimens in the study design and not a specific clinical setting with a lower number of cases. Interestingly, sensitivity was higher in non-hospitalized, even asymptomatic, than symptomatic hospitalized patients. Therefore, based on our findings, we suggested that the GeneFinder COVID-19 Ag Plus Rapid Test can be used as a screening test for early SARS-CoV-2 diagnosis to slow down virus spread. The RATs predicting negative value was 98.69% (95% CI: 97.17–99.40%). In line with previous reports [13,14,15,16,17,18,19,20,21,22,23,24,25,26,27,28,29], SARS-CoV-2 RNA load was significantly higher in RT-PCR+/RAT+ specimens than in RT-PCR+/RAT- samples. Of interest was how the size of the band and the intensity of the gold particles’ color could be used, not only to result in positivity but also to estimate viral load considering the Ct. It is worth mentioning here that RT-PCR results are inversely proportional with expression data. Therefore, it could be considered to have a tool that is easy to interpret the delta approaches. Although the Allplex™ 2019-nCoV Assay amplifies up to 40 cycles and states that any gene with Ct < 40 means positive results, there is increasing appreciation that Ct 30–35 is considered borderline [15]. Probably, Ct > 35 could reflect viral fragments since the virus cannot be isolated and cultured at this level of viral load. In our opinion and from COI data, the Ct 28–30 could be a suitable cut-point. Future insights are needed concerning single discordant cases related to cross-reactivity assayed with other respiratory viruses, including a more significant number of cases. However, surprisingly, the GeneFinder COVID-19 Ag Plus Rapid Test was able to recognize also the VOCs, either delta, different type of lineages and omicron, both BA.1, different lineages and BA.2 [12]. Our study represents the first report on the GeneFinder COVID-19 Ag Plus Rapid Test performance. The topic concerning the control of pandemic spread via self-monitoring is a need for the entire world [17,27,28,29]. A recent systematic review and meta-analysis pointed out that RATs detect the vast majority of SARS-CoV-2-infected persons within the first week of symptom onset and with high viral load [27]. Thus, they can have high utility for diagnostic purposes in the early phase of the infection. Indeed, RATs are a valuable tool to fight the spread of SARS-CoV-2. Therefore, RATs are increasingly being integrated in testing strategies worldwide [27]. Studies of the RATs have shown variable performance. Despite the low analytic sensitivity, RATs are inexpensive and can be used frequently to detect infected individuals who are symptomatic, pre-symptomatic and without known or suspected exposure to SARS-CoV-2. Thus, they can have high utility for diagnostic purposes in the early phase of the disease, making them a valuable tool to fight the spread of the infection [28,29]. We therefore conclude that screening should prioritize accessibility, frequency and sample-to-answer time; analytical limits of detection should be secondary, especially in a low-income country area in which laboratory testing capability is low [29,30,31,32,33].

## 5. Conclusions

The SARS-CoV-2 Real-Time RT-PCR has high specificity and sensitivity. However, the rapid assay for SARS-CoV-2 antigen detection (GeneFinder COVID-19 Ag Plus Rapid Test) showed excellent clinical sensitivity and specificity on asymptomatic subjects. More crucially, our data suggest that patients with Real-Time-RT-PCR-proven COVID-19 testing negative by RATs are unlikely to be infectious. Therefore, we point out that GeneFinder COVID-19 Ag Plus Rapid Test can be a valid screening assay, especially in a high prevalence area, even in the light that it can also recognize variants of concern.

## Figures and Tables

**Table 1:** NAAT vs. GeneFinder COVID-19 Ag Plus Rapid Test.

Test	RAT+	RAT−	Total
NAAT+	145	6 *	151
NAAT−	1 **	451	452
Total	146	457	603

* Six false-negative results were obtained from samples with RT-PCR cycle threshold Ct >28. ** In one case, we found a cross-reactivity result for RSV.

**Table 2:** GeneFinder COVID-19 Ag Plus Rapid Test performance, compared to RT-PCR standard assay (NAAT).

	Sensitivity	Specificity	Overall Percent Agreement	PPV	NPV	Cohen’s Kappa
	96.03%	99.78%	98.84%	99.32%	98.69%	96.87 (SE: 0.01)
Confidence Interval (CI 95%)	91.55–98.53%	98.77–99.99%	97.62–99.53%	95.43–99.90%	97.17–99.40%	0.9457–0.9918

**Table 3:** The overall sensitivity of the GeneFinder COVID-19 Ag Plus Rapid Test according to the N gene cycle threshold in SH, NH and ANH patients.

N Gene Cycle Threshold Value	SH (*n* = 23) Sensitivity	NH (*n* = 101) Sensitivity	ANH (*n* = 14) Sensitivity
<25	100%	100%	100%
25–30	100%	100%	100%
>30	100%	100%	-

**Table 4:** Variant of Concern tested on GeneFinder COVID-19 Ag Plus Rapid Test.

VOC	Clade	Lineage	N-Protein Mutations
Delta	21k	B.1.617.2	D63G; G215C; D377Y
Delta	21k	AY.4	D63G; R203M; G215C; D377Y
Delta	21J	AY.39	A35; D63G; R203M; G215C; D377Y
Delta	21K	AY.42	D63G; R203M; G215C; D377Y
Delta	21J	AY.122	A35; D63G; L139F; R203M; G215C; D377Y
Delta	21J	AY.46.6	D63G; R203M; G215C; D377Y
Delta	21A	AY.53	D63G; R203M; S327; D377Y; D399Y
Delta	21I	AY.61	D63G; T135I; R203M; D377Y
Delta	21I	AY.9.2	D63G; R203M; D377Y
Omicron	21k	B.1.1.529	P13L; GERS30G_del; R203K; G204R
Omicron	21k	BA.1	P13L; GERS30G_del; R203K; G204R
Omicron	21k	BA.1.1	P13L; GERS30G_del; R203K; G204R
Omicron	21L	BA.2	P13L; GERS30G_del; R203K; G204R; S413R
Omicron	21L	BA.2	P13L; GERS30G_del; R203K; G204R; S413R

## Data Availability

Data supporting reported results can be found at the Department of Microbiology and Virology, Pugliese-Ciaccio Hospital, Catanzaro, Italy and at Unit of Microbiology and Virology, North Health Center ASP 5, Reggio Calabria, Italy.
